# Does work-induced fatigue accumulate across three compressed 12 hour shifts in hospital nurses and aides?

**DOI:** 10.1371/journal.pone.0211715

**Published:** 2019-02-07

**Authors:** Brennan J. Thompson

**Affiliations:** 1 Department of Kinesiology and Health Science, Utah State University, Logan, Utah, United States of America; 2 Movement Research Suite, Sorenson Legacy Foundation Center for Clinical Excellence, Utah State University, Logan, Utah, United States of America; University of Auckland, NEW ZEALAND

## Abstract

Fatigue-related impairments in the nursing workforce contribute to a multitude of health, safety, and economic consequences at the individual, organizational and societal levels. Long and compressed work schedules are commonly worked in the healthcare industry, but more research is needed to understand the cumulative effects of multiple work shifts on physiology-based performance outcomes in nurses. The purpose of this study was to compare the effects of a single nursing work shift versus three compressed (one every 24 hours) 12 hour shifts on performance-based fatigue in nurses and aides. Twenty-six fulltime hospital working nurses and aides (age = 36.1 ± 13.3 years) reported to the lab for testing before, immediately after working a single 12 hour shift, and after working three 12 hour shifts in a 72 hour period. Outcome measures included vigilance-based reaction time, lapses of attention, and muscle function assessments (lower and upper body muscle strength, explosive strength and vertical jump performance). All variables except hand grip strength showed a significant decline following the three work shifts. The psychomotor vigilance reaction time and lapses of attention variables also generally showed a significant decline from the end of shift one to the end of shift three, indicting an accumulation of fatigue in these metrics with increasing number of shifts worked. Muscle function variables responded early in the duty cycle, showing a significant decline after a single work shift, but did no further decline by the end of the third shift. These findings use objective measures to substantiate that fatigue impairments occur from working a single 12 hour shift, and in several instances, increase further with more successive work shifts. Caution should be employed by personnel and administrators with work schedules involving multiple compressed 12 hour shifts. Fatigue management strategies may be used to improve risks and consequences from fatigue-related mishaps, and this study reports several variables that appear sensitive to identifying and tracking fatigue in this population.

## Introduction

The evidence regarding the prevalence and consequences of fatigue in the at-risk nursing population has resulted in an urgency for increased awareness and effective management plans. Recently, many respected professional organizations have put forward position statements and guidelines drawing awareness to healthcare worker fatigue and its consequences [1–5], with the overall aim of these organizations to legitimize fatigue as a serious and pressing issue. For instance, the American Nurses Association [6] clarified that nurses have an “ethical responsibility and duty to their patients to recognize their level of fatigue before accepting patient assignments.”

Much attention has recently been given to the impact of healthcare provider fatigue on patient outcomes. For example, the dangers of healthcare provider-based lapses on patient-related consequences are described by the recent findings of James [7] who reported that an estimated 400,000 deaths and 2 to 4 million nonlethal serious medical events per year are due to healthcare provider errors. These statistics are especially alarming considering that such errors are largely preventable given they have empirically been linked, at least in part, to the workers’ fatigue [8–12]. In addition to the greater injury risk to the fatigue-impaired worker [12, 13], fatigue of the healthcare provider is a primary contributor to negative patient outcomes, which includes poor quality of care [14], and increased adverse events such as medication errors due to reduced vigilance and attentional lapses [8, 14–16]. A range of consequences may result from fatigue in those who perform patient care tasks (nursing workers) which may directly result in a cascade of poor employee/organizational outcomes such as burnout, a multitude of caregiver health and safety problems, high turnover rates, staff shortages leading to a negative impact on the overall health of the organization (e.g., profitability) [17].

It is understood that workplace practices and policies can influence worker outcomes such as health, performance, work-related injury risks, and as already mentioned, patient safety and quality of care [9, 18–20]. Previous researchers have highlighted evidence suggesting that demanding schedules—which includes 12 hour shifts and compressed schedules typical to healthcare settings—are likely a major contributor toward these alarming injury statistics [9, 19, 21]. However, a bulk of the research investigating work-related influences on healthcare worker health outcomes has focused on the effects of a single work shift, with the primary interest being on the length of the shift (e.g., 8 versus 12 hour shifts) [21]. Unfortunately, this has left a large body of knowledge void regarding the effects of successive shifts (cumulative shifts in a row) on health, performance, and ultimately injury responses in this important but vulnerable nursing worker population. For example, Hopcia et al. [21] have suggested that recent trends of consecutive work shifts leading to cumulative hours may impact injury risks, and concluded that many studies have examined overtime work patterns, but have inadequately evaluated the influence of increasing shifts and hours worked in a given time period on worker outcomes (e.g., fatigue).

Manifestation of high a prevalence of fatigue in the working population [22] has spawned growing concern, particularly because of the apparent link to reduced performance, high sickness absence, and work disability [23, 24]. This is especially detrimental for patient care providers (nurses) who require uncompromised levels of accuracy throughout the duration of the work period [9]. Consequences of error in this profession are at a maximum and are poised to spread a ripple effect of negative impact across individuals and organizations. For instance, even minor impairments in a nurse’s performance, such as lapses in attention, judgment or responsiveness, could lead to disastrous patient outcomes, as well as an increased risk for adversely impacting the nurse’s mental and physical health and well-being (the occurrence of which is supported by the high injury rates and burnout prevalence in this working population). Thus, these nurse-originating consequences have the potential to affect persons on multiple levels as well as the micro and macro organizational structures (i.e. floor/unit, hospital, healthcare system) in which they function. Demographic and job-specific predisposition to this profession only exacerbates the problem as female gender (>90% of nurses), low decision authority, and long and compressed work schedules—all proportionally high in healthcare occupations—are risk factors for fatigue and predictors of long-term sickness absence [23]. However, despite its prevalence and consequences, it is surprising that an objective physical manifestation of fatigue has not been well documented in nursing workers. Occupational specific fatigue research has almost exclusively implemented subjective assessments in the form of questionnaires [25]; being largely based upon self-reports dealing with perceived fatigue and outcomes based upon work-related incidents [13]. Such subjectivity is not representative of actual human performance-based functionality, and moreover, can easily be manipulated by the employee to reflect the desired outcome. Thus, the need for more objective, scientifically valid indicators of fatigue in nursing workers is critical to enhance the understanding of fatigue risk factors and predisposing employee characteristics, and to utilize such information as a fatigue management tool [9] to help improve nurse, patient, and healthcare facility outcomes.

A common concept in the literature concerns the total amount of exposure for risk factors [26], and a number of studies have demonstrated that a predominant factor for musculoskeletal injuries involves high physical work demands over the period of a shift [27–29]. The total yield of exposure is thus influenced by the factors of high workload demands (intensity of workload), and long and frequent work shifts. One aspect of exposure that has received little research attention is the frequency of the work shifts. A recent study by our laboratory is among the very few that has examined the effects of multiple work shifts on nurses performance outcomes using objective assessments (reaction time, balance etc.). In that study, we showed that a compressed work schedule yielded significant declines in various performance measures. However, that study was not able to compare the effects of the work shifts in a “dose response” manner. In other words, it remains to be determined whether these fatigue related deficits were present during the early portion of the multiple shift work cycle (e.g. after a single work shift). It may be presumed, according to the exposure model of work risk factors [30], that performance-based fatigue may increase cumulatively across multiple compressed shifts. This scenario would render the risks of fatigue in the nurse worker proportionally more impactful toward the end of the multiple work shift period. However, elucidating these effects requires fatigue assessments to be performed at both the early and later time periods of the multiple day work schedule. Thus, the purpose of the present investigation was to examine the effects of a single nursing work shift versus three successive (e.g., three shifts in a 72 hour period) 12 hour shifts on performance based fatigue in nurses and aides using vigilance-based reaction time, lapses of attention and muscle function assessments as objective performance measures. We hypothesized that there would be greater impairments in all the performance measures following the third shift compared to the single work shift due to an accumulating effect of fatigue from the increasing work exposure across the compressed multiple work shift period.

## Materials and methods

### Participants

The study used a convenience sampling technique to recruit nurses who were employed at local hospitals (≤ 25 miles of the university). The nurses were solicited from local hospitals via advertisements, flyers, and word of mouth and interested persons were prescreened according to the eligibility criteria (below) prior to reporting to the lab and/or being enrolled in the study. Twenty-six nurses and aides (mean ± SD: age = 36.1 ± 13.3 [range = 18–62] years, body mass = 77.7 ± 20.3 kg, height = 166.0 ± 5.7 cm) volunteered for the investigation. Eligibility criteria required the healthcare workers to be, 1) a currently working registered nurse (RN), licensed practical nurse (LPN) or certified nursing assistant (CNA), 2) working fulltime (36 hours per week) and 12 hour shifts, and 3) between the ages of 18–65 years. In addition, participants were required to be free of any neuromuscular diseases (e.g., Parkinson’s, multiple sclerosis), medically diagnosed sleep disorders, had no previous musculoskeletal injuries or surgery on their dominant leg within the previous 1 year, and could not be pregnant. Following screening and debriefing procedures, participants signed an informed consent document. This investigation and its methods were approved by the Utah State University Institutional Review Board (protocol # 7179).

### Experimental procedures

In order to investigate the effects of cumulative work shifts on fatigue, the study required participants to work three, 12 hour work shifts in a row. The testing protocol involved participants visiting the laboratory for testing on four separate occasions with the first visit involving study protocol debriefing, work schedule confirmation, and formally familiarizing the participants on the performance tests. The second visit was the pretest (Pre) and was scheduled within 24 hours of the first work shift and a minimum of 48 hours following any previous work shift in order to avoid the effects of previous work-induced fatigue on the testing measures [31]. The third visit occurred immediately following (i.e., within 1 hour of finishing the shift to allow for travel time) the first work shift (Mid1) and the fourth visit immediately followed the third and final work shift (Post). To help control for outside physical activity factors, participants were instructed to refrain from any structured exercise or vigorous physical activity during the course of the study (e.g., no structured exercise, competitive sports participation, or physically rigorous leisure activities that may induce muscle soreness or excessive fatigue).

### Assessments and measures

#### Reaction time

The psychomotor vigilance task (PVT) procedures used in this investigation followed the same protocol as has been detailed in our previous study on nurses [32]. Briefly, the PC-PVT platform was used as developed and validated by Khitrov et al. [33]. The test was administred using a PC (HP Z220, HP Inc., Palo Alto, CA) and an optical gaming mouse (Razer Abyssus, Razer, Carlsbad, CA). The PVT was 5 minutes in duration, and during the test participants were asked to turn off all personal electronic devices and were not allowed to have any other persons present in the room during the test administration [32]. PVT variables included the mean and slowest 10% reaction times as well as the number of minor lapses (defined as response times of between 500–1000 ms).

#### Vertical jump

Following the PVT, participants performed three maximal effort countermovement jumps on a jump mat (Just Jump Technologies, Huntsville, AL) in accordance to the procedures of Palmer et al. [34]. Briefly, participants placed both of their feet shoulder width apart on the jump mat with their hands positioned on their hips. The movement involved a rapid descending squat motion followed by an explosive ascending take-off. Participants were not allowed to take any steps prior to the jump and had to land with both feet on the mat. The jump mat estimates jump height (JH, cm) based on flight time, which is defined as the time that elapses from when the feet leave the mat until landing [34].

#### Strength assessments

Following the CMJs, isometric strength assessments were performed on the knee extensor, knee flexor, and wrist flexor (i.e., hand grip) muscle groups. For the hand grip test, three maximal voluntary contractions (MVCs) were performed on the dominant hand using a calibrated hydraulic hand dynamometer (Jamar 5030J1, Performance Health, Warrenville, IL). Five minutes following the hand grip MVCs, isometric knee extensions and flexions were performed on a calibrated dynamometer (Biodex System 3; Biodex Medical Systems, Shirley, NY). Participants were seated with restraining straps placed over the trunk, pelvis, and thigh, and the input axis of the dynamometer was aligned with the axis of rotation of the knee. Before the MVCs, participants performed a warm-up protocol of 10 submaximal extension and flexion contractions at a velocity of 120 deg·s^-1^. Isometric MVCs were performed for the knee extensors and flexors in random order, with 3 MVCs performed for each muscle group. The leg angles for the MVCs were set at 60 and 30° below the horizontal plane for the knee extensors and flexors, respectively [35]. The MVCs were 4 seconds in duration and a 2 minute rest period was provided between all MVCs. Participants received strong verbal encouragement and were instructed to “push”, “pull”, or “squeeze” as “hard and fast as possible for 4 seconds.”

The raw torque signal was sampled at 2 KHz with a Biopac data acquisition system (MP150WSW, Biopac Systems Inc., Santa Barbara, CA) and subsequently processed offline with custom written software (LabVIEW 2016, National Instruments, Austin, TX). The signals were scaled to units (Nm), gravity-corrected for limb weight (baseline was set to zero), and filtered with a fourth-order, zero phase-shift, low-pass Butterworth filter with a 50 Hz cutoff [36]. Peak torque (PT, Nm) was calculated as the highest 500 ms epoch during the MVC and rate of torque development (RTD100, Nm·s^-1^) was quantified as the linear slope of the ascending portion of the torque-time curve at 100 ms from onset. Signal onset was determined manually by an experienced investigator via visual inspection as the point where the signal first deflected from baseline [37]. Composite strength variables were also calculated by summing the values of the knee extensors and flexors muscle groups for each variable (PT and RTD).

### Statistical analyses

Repeated measures analyses of variance (ANOVAs) were used to examine differences across test trials (Pre vs. Mid1 vs. Post) in the performance variables. Bonferroni adjusted pairwise comparisons were used for post hoc comparisons. Cohen’s *d* statistics were also used to examine the effect size for mean differences between test trials with values of 0.20, 0.50, and 0.80 being considered as small, medium, and large, respectively. IBM SPSS Statistics (Version 25.0, SPSS Inc., Chicago, IL) was used for the statistical analyses, and an alpha level of P < 0.05 was considered statistically significant for all comparisons.

## Results

All participants had been working in their current job (hospital, floor, shift type) for at least 3 months prior to the study, and the mean time at their current job for all participants was 3.4 years. The study sample was comprised of 16 RNs, 8 CNAs, and 2 LPNs, and the mean ± SD total hours worked over the three shifts was 37.5 ± 1.7 hours (range of 34–41.5 hours). During the three day work schedule, all participants worked at least 11.5 hours and not more than 14 hours for each work shift completed in the study.

**Fig 1:**
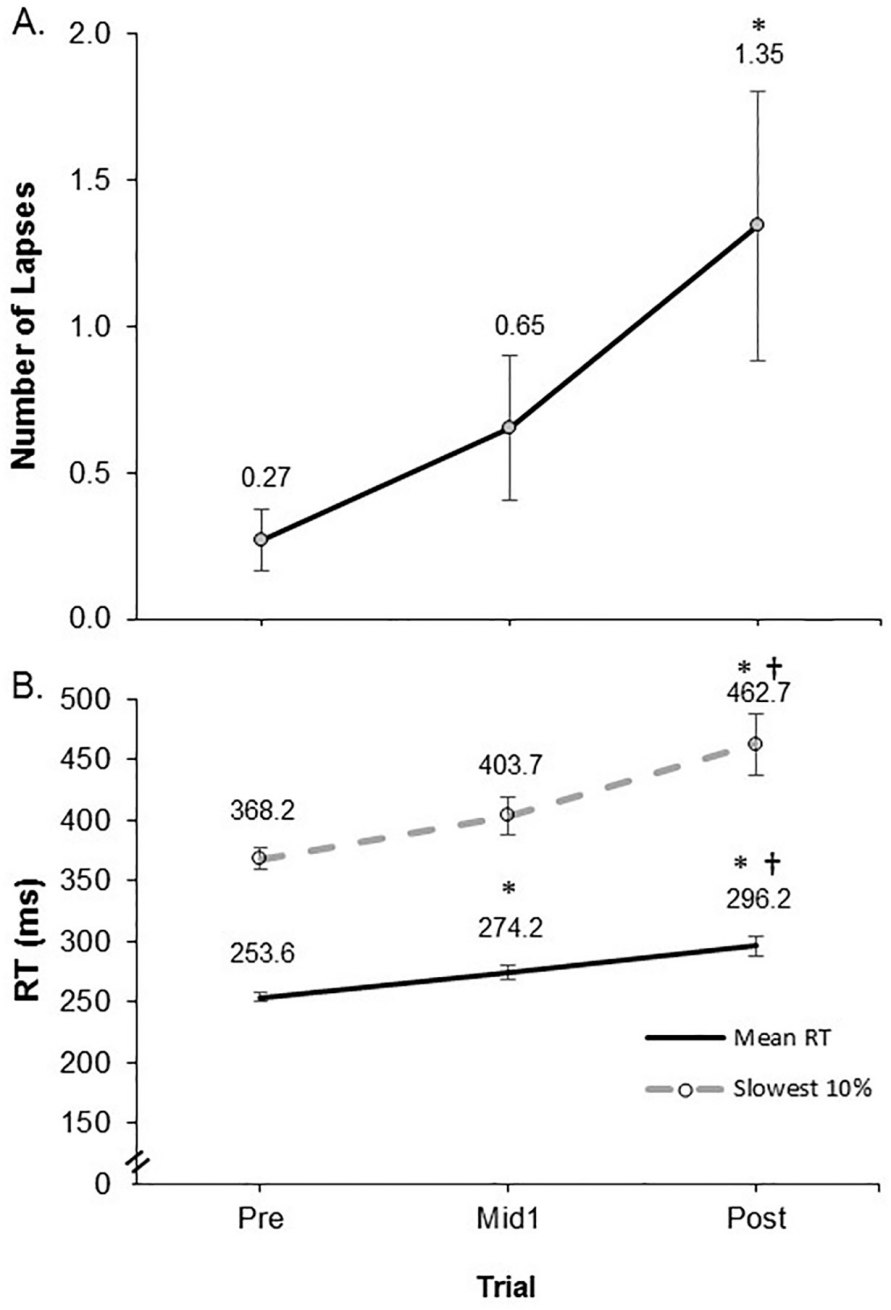
A) Lapses of attention, and B) mean and slowest 10% reaction time (RT) on the psychomotor vigilance task (PVT) before (Pre), after one 12 hour work shift (Mid1), and at the end (Post) of a consecutive three day nursing work shift schedule. Values are mean ± SE. * Significantly different from Pre and † Significantly different from Mid 1 at P < 0.05.

**Fig 2:**
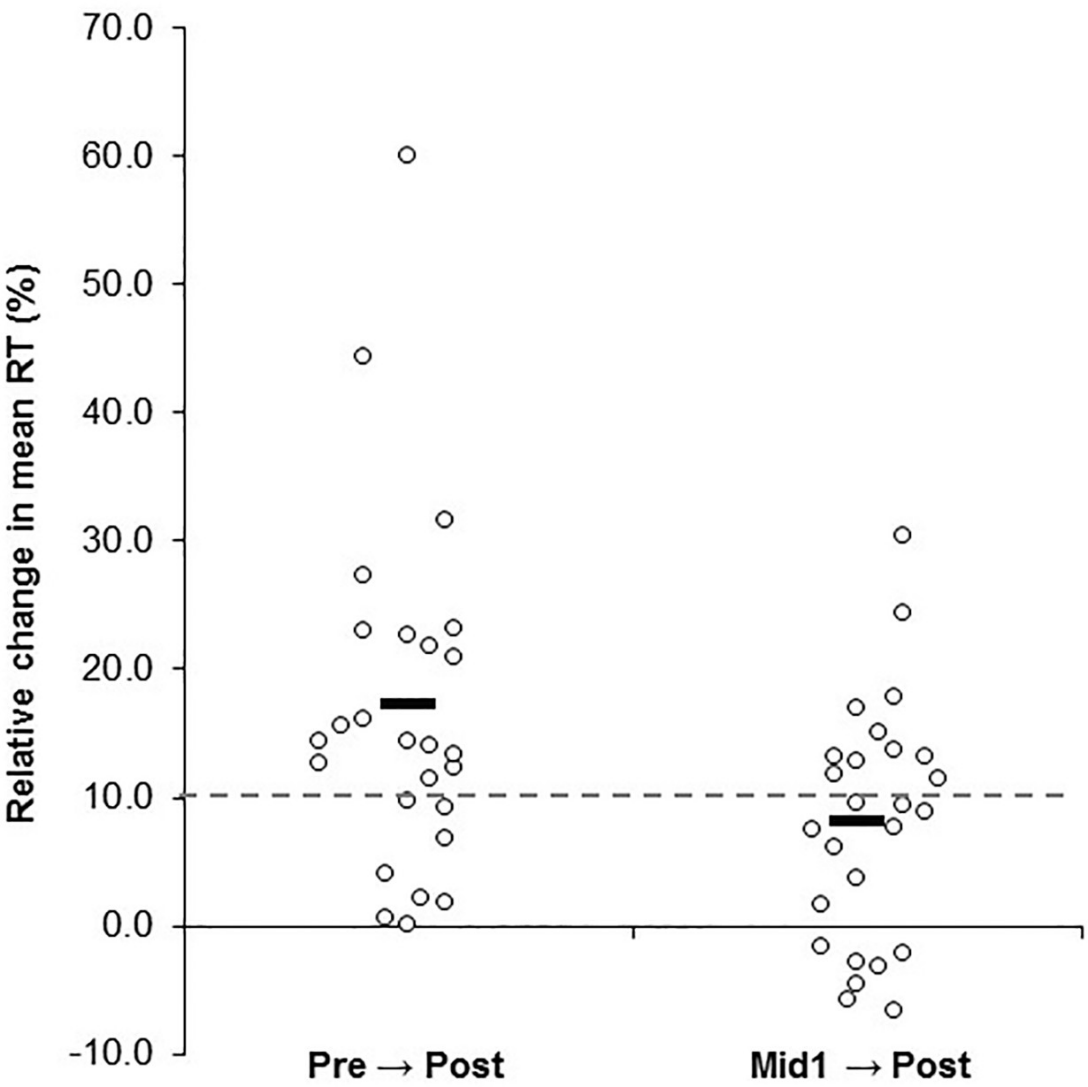
Individual scatter plot showing the relative (%) change scores for mean reaction time (RT) for each participant from baseline (Pre) to after the three consecutive work shifts (Post), and from the end of the first work shift (Mid1) to the end of the third shift (Post). Note, positive % change indicates poorer RT performance (i.e., an elongated response time versus earlier time trial). Horizontal dashed line represents a 10% reduced performance and is used here as an illustration of a hypothetical threshold where such a performance impairment may begin to negatively impact on-the-job task performance.

Fig 1 shows the PVT variables at Pre, Mid1, and Post three day work period. There was a main effect for time trial (P = 0.02) for the number of lapses of attention. Post hoc comparisons revealed that Post had a greater number of lapses compared to Pre (P = 0.04), whereas Mid1 was not different compared to Pre (P = 0.29) or Post (P = 0.22) (Fig 1A). There were main effects (P < 0.001) for time trial for both the mean RT and the slowest 10% RT variables. Post hoc comparisons revealed that for mean RT, Post was greater versus both Pre (P < 0.001) and Mid1 (P = 0.001) and Mid1 was greater than Pre (P < 0.001); whereas for slowest 10% RT, Post was greater than Pre (P < 0.01) and Mid1 (P = 0.03), but Mid1 was not statistically greater than Pre (P = 0.06) (Fig 1B). Fig 2 shows the individual relative (%) change scores in mean RT prom Pre to Post and from Mid1 to Post time trials.

**Fig 3:**
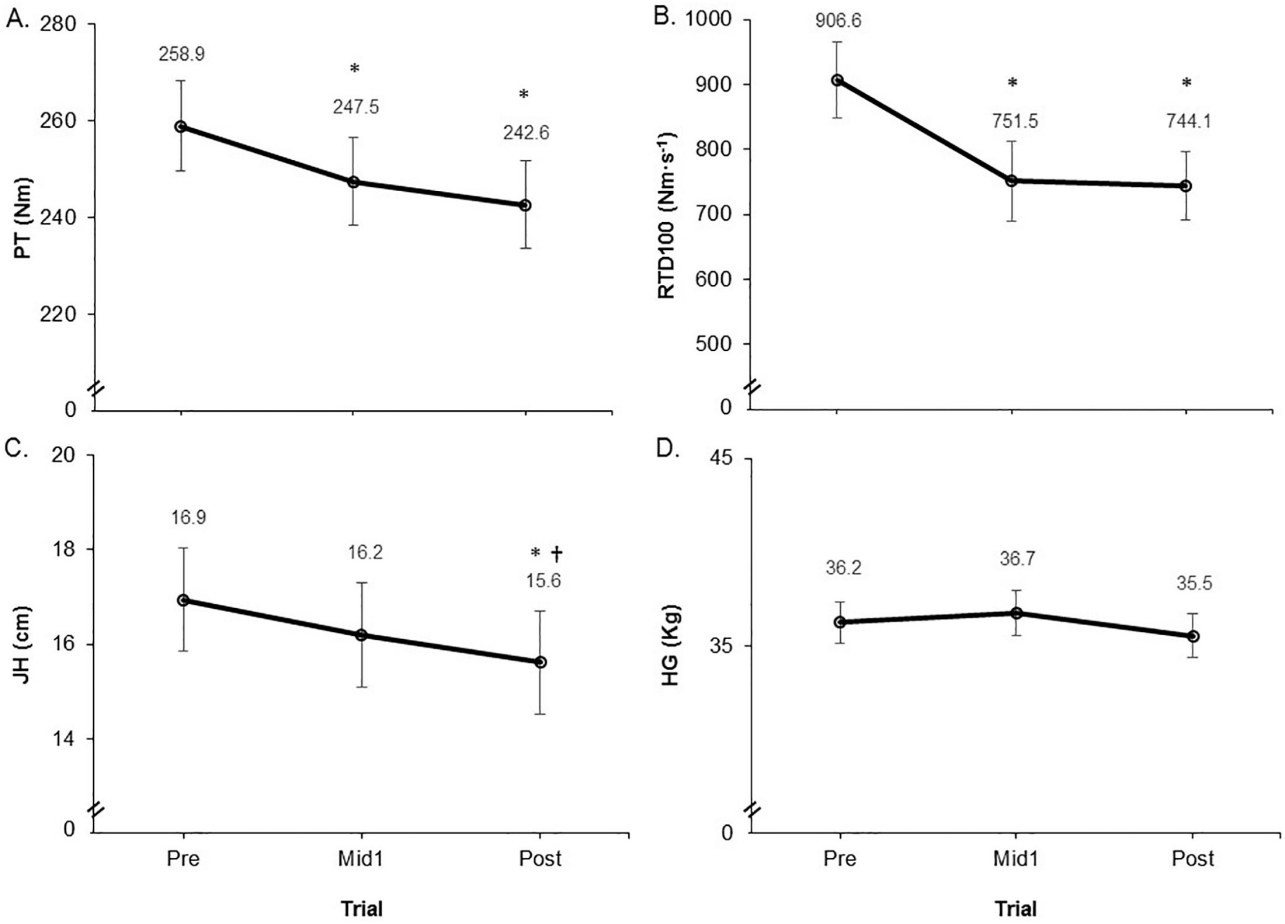
A) and B) are composite values (sum of the knee extensors and flexors) for the peak torque (PT) and rate of torque development (RTD) isometric strength variables, respectively, and C) and D) show countermovement jump height (JH) and hand grip (HG) strength, respectively, before (Pre), after one 12 hour work shift (Mid1), and at the end (Post) of a consecutive three day nursing work shift schedule. Values are mean ± SE. * Significantly different from Pre and † Significantly different from Mid1 at P < 0.05.

For isometric PT, there were main effects for time trial for the knee extensors (P = 0.03) and flexors (P = 0.03). Post hoc comparisons did not reach statistical significance for the knee extensors for any of the pairwise comparisons (P = 0.08–0.13; 174.6 ± 36.1, 167.8 ± 33.5, 162.8 ± 34.1 Nm, for Pre, Mid1 and Post, respectively), however, the knee flexors’ PT was reduced for the Mid1 compared to Pre (P = 0.05; 84.3 ± 16.3, 79.6 ± 17.0, 79.8 ± 15.8 Nm). There was a main effect for time trial for the composite PT variable (P = 0.01) with post hoc comparisons revealing reduced PT for Mid1 (P = 0.03) and Post (P = 0.05) versus Pre (Fig 3A).

For RTD100, there were main effects for time trial for the knee extensors (P = 0.01) and flexors (P < 0.01). Post hoc comparisons for knee extensors and flexors revealed that Mid1 (P = 0.02 and 0.03, extensors and flexors, respectively) and Post (P = 0.01 and 0.03) were reduced compared to Pre, but there were no differences between Mid1 and Post (P = 1.0; extensors = 652.0 ± 250.6, 543.8 ± 248.4, 542.4 ± 237.5 Nm·s^-1^ and flexors = 254.6 ± 104.9, 207.7 ± 92.1, 201.7 ± 81.0 Nm·s^-1^, for Pre, Mid1, and Post, respectively). There was a main effect for time trial for the composite RTD100 variable (P < 0.001) with post hoc comparisons revealing reduced RTD100 for Mid1 (P = 0.001) and Post (P < 0.001) versus Pre, but no differences between Mid1 and Post (P = 1.0) (Fig 3B).

**Fig 4:**
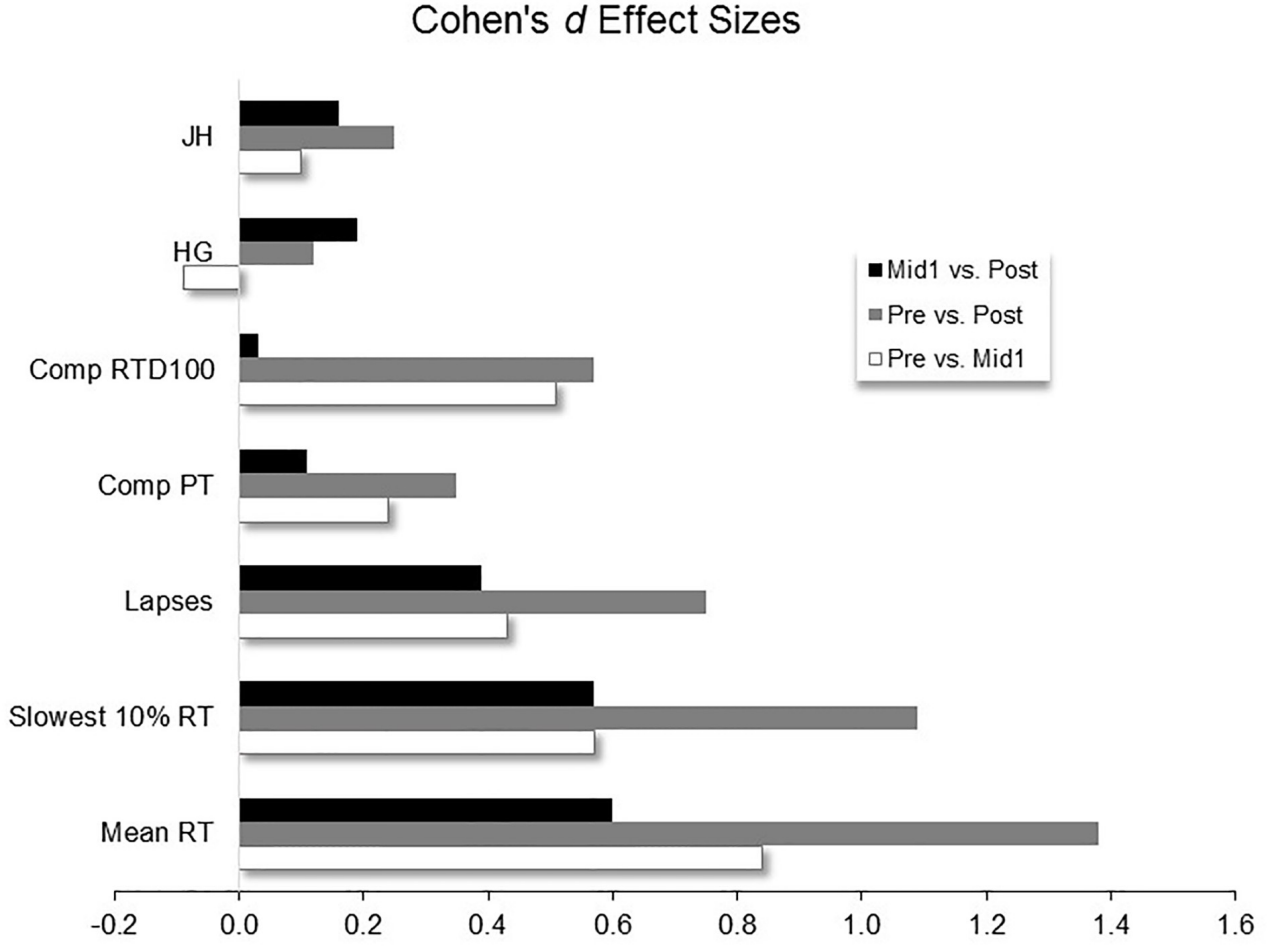
Cohen’s *d* effect sizes comparing time trial mean differences on primary outcome measures before (Pre), after one 12 hour work shift (Mid1), and at the end (Post) of the three day work shift schedule. JH = Jump Height; HG = Hand Grip; Comp = Composite score of knee extensors and flexors muscle groups; PT = Peak Torque; RTD100 = Rate of Torque Development at 100 ms; RT = Reaction Time. Note, positive effect size values indicate poorer performance on each variable when compared to the earlier time trial (e.g., reduced jump height, composite peak torque etc. or increased reaction times, lapses etc.).

There was no main effect for HG strength (P = 0.42; Fig 3D), but there was a main effect for JH (P = 0.01). Post hoc comparisons revealed that JH was reduced for Post versus both Pre (P < 0.01) and Mid1 (P = 0.04), but there were no differences between Pre and Mid1 (P = 0.87) (Fig 3C). The effect sizes comparing mean differences between time trials for the primary outcome measures are shown in Fig 4.

## Discussion

The present investigation showed that fatigue-based impairments in various performance tasks were observed after a single 12 hour work shift, and that for some tasks, these impairments were more exacerbated following multiple (three successive) work shifts.

Collectively, the changes in the PVT variables showed moderate and large effects induced from the work shifts (Fig 4). For mean RT, the impairments on reaction time responded in an incremental pattern with an increased number of shifts worked. For example, the mean RT was impaired by 8% following work shift one, and by 17% after three shifts. Indeed, the largest effect for all variables was for the mean RT variable following three work shifts (ES = 1.40), however, it is notable that the single work shift also showed a large effect (ES = 0.85). Moreover, the ES of 0.60 from the end of the single work shift (Mid1) to the end of the third shift (Post) reveals a moderately large effect as a result of working two more successive shifts after the first shift. The present finding of impaired RT following a 12 hour nursing work shift is not in agreement with the findings of Surani et al. [25] who reported no changes in PVT RT or lapses of attention in nurses after working a single 12 hour shift. The reasons for the discrepancy are unclear, however, the testing designs used between the studies may in part explain the inconsistency. Surani et al. [25] tested nurses at the start and end of the work shift, whereas the present study tested the baseline PVT at the same time of day (as the follow up test) on the day prior to the first work shift. This studies’ test design attempted to control for any possible testing effect due to the time of day which the test was administered. The prospective cohort study design used in these studies (i.e., no true control group) includes the possibility that the within-day, before-after shift test design could be influenced by time of day test specific characteristics (which are presently unknown). Therefore, controlling for a time of day test effect by testing the pretest at the same time of day as the follow up test may better enable the test to capture changes that may have occurred from the experiment and not from daily circadian fluctuations in biological processes which affect arousal/mood states across the day (e.g., a pretest early in the morning and posttest performed in the evening may have a test specific effect for the PVT, especially given that it is an arousal/mood-based assessment). Thus, the present data shows that impaired vigilance-based RT does occur from a single 12 hour work shift in a population of nurses, although more work is needed to substantiate these results.

There is a conspicuous void in the literature investigating multiple work shifts (as opposed to a single work shift) and performance impairments—which limits our ability to experimentally compare these findings with previous work. However, the present findings of impaired RT following multiple work shifts generally supports the results of Thompson et al. [32] who recently showed that PVT RT was impaired by 6–11% following a compressed (but not successive) 12 hour work shift schedule in a similar population of nurses using identical PVT test procedures. The previous Thompson et al. study [32] examined three, 12 hour shifts worked in a four day period (with one day off in the middle), versus the three day in a row shift schedule investigated presently. A comparison of these studies shows that both types of compressed schedules (three shifts out of four, and three in a row) led to impaired PVT performance at the end of the multiple work shift cycles, but that the series of three shifts in a row schedule implemented in the current study induced greater impairments. For instance, the slowest 10% RT variable was impaired 25% in the current study using the three shifts in a row schedule, versus 11% in the previous study [32] which had a shift off in the middle of the multiple work day schedule. Also, the mean RT ES of 0.48 in the earlier study [32] was considerably smaller compared to the large ES of 0.84 reported in this study.

Another key finding pertaining to the PVT performance was the substantial increase in lapses of attention that occurred as the work shift numbers increased. In this study, three successive 12 hour nursing work shifts resulted in a five fold increase in the number of lapses of attention during the PVT (Fig 1). There was also a moderate effect observed for an increase in lapses of attention following the three shifts compared to only one work shift (mean lapses were 1.35 vs. 0.65 for three shifts versus one shift, respectively; ES Mid1 –Post = 0.39). Thompson et al. [32] also found an increase in lapses of attention following the multiple nursing work shifts examined in that study, for which it was shown that a non-consecutive multiple shift nursing work period increased PVT lapses of attention from 0.25 to 0.72 –a 3 fold increase in lapses of attention for that particular type of compressed schedule. It is noteworthy that each type of 12 hour shift pattern (i.e., one shift, three shifts in four days, and three shifts in three days) showed a tendency to induce a greater number of lapses of attention, demonstrating that apparently almost any 12 hour nursing work period may potentially pose a risk for increasing fatigue related lapses of attention. However, the present findings highlight that the three successive work shift schedule induced disproportionately greater impairments in attention lapses compared to either a single shift, or a multiple work shift pattern with one day of recovery allotted in the middle of the schedule. The severity of the impact reported here on attention lapses resulting from three successive 12 hour work shifts (in 72 hours) is concerning as such effects from this specific type of demanding schedule may incur substantial risks for medical errors, accidents and injuries. In particular, this study provides support for the accumulating detrimental effects of multiple, successive 12 hour shifts, because the present data increases the ability to compare multiple types of 12 hour work schedules which have examined PVT-based fatigue outcomes in nurses. Drawing on these comparisons has revealed that the three, 12 hour shift schedule in 72 hours may largely increase risks associated with psychomotor vigilance-based fatigue, and provides some substantive evidence that these specific schedules should be used with caution. However, it still remains unclear as to what extent certain work or personal factors influence the magnitude of these deleterious work imposed responses—such as the type of shift or unit (e.g., rotating versus day shifts, ER versus labor and delivery units etc.) worked [25] or what personal attributes (physical or psychological factors) may contribute to a given nurse worker being susceptible for a greater fatigue risk from a challenging compressed work shift schedule.

Maximal and explosive lower body strength were significantly reduced at both Mid1 and Post. It is interesting to note that both lower body strength-related variables were reduced after a single work shift and tended to show a similar magnitude of impairment following the third successive shift (Post) versus the single shift. This study design does not allow the determination of the level of recovery of these variables between each or any of the work shifts. It therefore is not feasible to determine if these variables had remained reduced from the end of the first work shift on through to the end of the three day work period, or if they had recovered during some point during that period but were undergoing a reduction at the end of each work shift. If these variables were reduced during the entire work period following the first shift (without recovery) this scenario would be particularly concerning as this would indicate that there was a reduction in lower body muscle function that persisted across almost the entire multiple work day period. Such a constant impairment would result in increased risk for injuries and/or a reduced ability to perform work tasks (e.g. ability to recover from a slip or trip, or lift or move a patient safely etc.) during a large portion of the multiple work shift schedule. It is important to note that the RTD100 variable showed a greater work-induced reduction compared to PT (18% versus 6.3% reduction for RTD100 and PT from Pre to Post, respectively). This could be impactful on nurses’ work performance and safety because the RTD variable is perhaps the most important muscle function characteristic for successfully performing many important physical tasks that may be important to nursing work, such as maintaining one’s postural balance [38], preventing falls [39] and enhanced functional performance abilities [37, 40, 41]. Based on these findings, which are in agreement with our earlier work showing nurses’ work-induced fatigue significantly affects RTD [31], it appears that the RTD variable is a sensitive marker of work-induced fatigue in nurses for the lower body performance. Thus, using RTD as a measure capable of identifying fatigue in nurses may be an intriguing variable to study in future work to determine if it may be useful in practice as a means to objectively monitor fatigue levels in order to help address a worker’s “fitness-for-duty”. Lastly, hand grip strength was not impaired as a result of working either one or three nursing work shifts. This may suggest that the upper limb may become less fatigued when compared to the lower limbs as a result of the types of work tasks typically performed during nursing work. For example, the majority of the more fatiguing physical demands performed during nurses’ work duties are tasks that predominantly involve the lower body, such as a high proportion of time committed to standing, walking, lifting activities etc. [42]. Thus, based on the present results, the hand grip strength peak force variable is not recommended for identifying or monitoring fatigue in nursing workers, as this measure may not capture the specificity of fatigue that may be present due to the task demands typical of most nursing work duties.

Jump height showed a relatively small (ES = 0.25) but statistically significant reduction following three successive work shifts. Our previous study [31] examining work-induced fatigue in nurses showed that jump height did not change following three compressed but non-consecutive 12 hour work shifts, despite maximal (PT) and explosive (RTD) strength variables being significantly reduced. The slight reduction of this measure in the present study is probably due to the more demanding (three successive shifts) work schedule performed in this study. Nevertheless, when taken together, this variable does not appear to be very sensitive to nursing work-induced fatigue. The plausible reasons for this have been detailed previously [31]. Briefly, the explanation may be due to the distinct mechanisms inherent to the countermovement jump which include a dynamically loaded prestretch that elicits stored elastic energy and neural reflex responses representing physiological mechanisms that may mask basic neuromuscular fatigue. Moreover, as the jump height test is a measure of one’s maximal jump capacity during a single attempt, we should consider the possibility that this may not capture true underlying fatigue due to the potential ability of a motivated person to very briefly achieve a high performance if required for only a single maximal attempt (i.e., an exceptional performance could theoretically still be achieved under conditions of fatigue, if it only required one exemplary effort). Along these lines, it would be intriguing to investigate whether physical tests which require a more repetitive performance routine are more sensitive to identifying underlying fatigue, because such a test may not allow the ability to overcome the underlying fatigue with a single attempt. Because of the need for accurate, objective fatigue identification assessments in fatigue-prone work forces, more research work needs to be done examining other potentially sensitive physical tests for identifying work-related fatigue. One such test may include a global lower body functional test that could be easily executed in the field (key features of the vertical jump) but performed with a repetitive routine, such as averaging the jump height over 10 or 15 repetitions, as a possible means to enhance the tests sensitivity to fatigue.

There were some potential limitations of the current study. The lack of a control group prohibits the detection of any possible baseline bias. However, to counteract this, controls were employed that required at least 2 days off work, as well as the requirement of no exercise or vigorous physical activity within the 2 days prior to the pretest in order to help control for differences in baseline status among nurses. In addition, the present study sample included only females specifically working 12 hour shifts from a small sample of hospitals in Northern Utah, which may limit the applicability of the findings to other healthcare worker populations (e.g., male nurses, 8-hour work shift lengths etc.).

## Conclusions

The demands of three consecutive (day-to-day) 12 hour nursing work shifts led to impairments in reaction time, greater lapses of attention reductions in multiple measures of muscle function. A particular novel finding was that vigilance-based reaction time measures showed an accumulating impairment progressively worsening from a single 12 hour shift to the end of the third shift. Several muscle function assessments also showed sensitivity to nursing work fatigue, and these were impaired early on in the work cycle such that they were significantly reduced after only a single 12 hour shift. This study provides a basis for describing fatigue characteristics in nursing workers across multiple shifts, however, future studies should build from this work and include longer work shift and, if possible, more fatigue assessment time points to help determine when fatigue accumulation becomes the most severe, and at what point recovery strategies may most effectively be applied across a nurses work schedule in order to reduce the heavy risks and burdens associated with fatigue-induced impairments in performance. These findings demonstrate that fatigue is objectively substantiated in nurses working 12 hour shifts. Thus, nursing staff, managers, and administrators may consider using fatigue management strategies as a countermeasure to the safety consequences associated with nurse fatigue. In particular, our work in this area provides support for the use of the computer based PVT as a useful and sensitive test for identifying/tracking fatigue in this specific population, and further work could develop more mobile muscle function assessments in order to improve the feasibility of using the rate of torque development variable as another potentially useful indicator of impairments in lower body muscle function.

## Supporting information

S1 Table Values of the peak torque, rate of torque development, grip strength, jump height and PVT-based reaction time and lapses of attention measures for each of the three testing sessions. (XLSX)
